# Genetic diversity of *Salmonella* Paratyphi A isolated from enteric fever patients in Bangladesh from 2008 to 2018

**DOI:** 10.1371/journal.pntd.0009748

**Published:** 2021-10-14

**Authors:** Sadia Isfat Ara Rahman, To Nguyen Thi Nguyen, Farhana Khanam, Nicholas R. Thomson, Zoe A. Dyson, Alyce Taylor-Brown, Emran Kabir Chowdhury, Gordon Dougan, Stephen Baker, Firdausi Qadri

**Affiliations:** 1 Infectious Diseases Division, International Centre for Diarrheal Disease Research, Bangladesh (icddr,b), Dhaka, Bangladesh; 2 Oxford University Clinical Research Unit, The Hospital for Tropical Diseases, Ho Chi Minh City, Vietnam; 3 Wellcome Sanger Institute, Wellcome Genome Campus, Hinxton, Cambridge, United Kingdom; 4 Department of Infection Biology, Faculty of Infections and Tropical Diseases, London School of Hygiene and Tropical Medicine, London, United Kingdom; 5 Department of Medicine, University of Cambridge School of Clinical Medicine, Cambridge Biomedical Campus, Cambridge, United Kingdom; 6 Department of Infectious Diseases, Central Clinical School, Monash University, Melbourne, Victoria, Australia; 7 Department of Biochemistry and Molecular Biology, University of Dhaka, Dhaka, Bangladesh; 8 University of Cambridge School of Clinical Medicine, Cambridge Biomedical Campus, Cambridge, United Kingdom; Mohammed Bin Rashid University of Medicine and Health Sciences, UNITED ARAB EMIRATES

## Abstract

**Background:**

The proportion of enteric fever cases caused by *Salmonella* Paratyphi A is increasing and may increase further as we begin to introduce typhoid conjugate vaccines (TCVs*)*. While numerous epidemiological and genomic studies have been conducted for *S*. Typhi, there are limited data describing the genomic epidemiology of *S*. Paratyphi A in especially in endemic settings, such as Bangladesh.

**Principal findings:**

We conducted whole genome sequencing (WGS) of 67 *S*. Paratyphi A isolated between 2008 and 2018 from eight enteric disease surveillance sites across Bangladesh. We performed a detailed phylogenetic analysis of these sequence data incorporating sequences from 242 previously sequenced *S*. Paratyphi A isolates from a global collection and provided evidence of lineage migration from neighboring countries in South Asia. The data revealed that the majority of the Bangladeshi *S*. Paratyphi A isolates belonged to the dominant global lineage A (67.2%), while the remainder were either lineage C (19.4%) or F (13.4%). The population structure was relatively homogenous across the country as we did not find any significant lineage distributions between study sites inside or outside Dhaka. Our genomic data showed presence of single point mutations in *gyrA* gene either at codon 83 or 87 associated with decreased fluoroquinolone susceptibility in all Bangladeshi *S*. Paratyphi A isolates. Notably, we identified the pHCM2- like cryptic plasmid which was highly similar to *S*. Typhi plasmids circulating in Bangladesh and has not been previously identified in *S*. Paratyphi A organisms.

**Significance:**

This study demonstrates the utility of WGS to monitor the ongoing evolution of this emerging enteric pathogen. Novel insights into the genetic structure of *S*. Paratyphi A will aid the understanding of both regional and global circulation patterns of this emerging pathogen and provide a framework for future genomic surveillance studies.

## Author summary

*Salmonella enterica* serovar Paratyphi A is an understudied cause of enteric fever which has started replacing *Salmonella* Typhi in some endemic regions. Currently, there are limited genomic epidemiology data to understand the true disease burden of disease caused by *S*. Paratyphi A in poor resource settings like Bangladesh. Our genomic data revealed that the population structure of *S*. Paratyphi A in Bangladesh comprised of only previously defined lineages A, C and F and carried single point mutation in *gyrA* gene associated with decreased fluoroquinolone susceptibility with no evidence of multi-drug resistance. Additionally, the global context revealed the clustering of organisms associated with neighboring countries in South Asia. We identified the pHCM2-like cryptic plasmid among our collection, which was not found in any *S*. Paratyphi A isolates previously. A comparative plasmid analysis showed that *S*. Paratyphi A pHCM2-like plasmid was highly similar to the plasmid found in *S*. Typhi but significantly different to a similar plasmid from non-invasive *Salmonella* Paratyphi B variant Java isolate from Bangladesh. This present study highlights the importance of sustained genomic surveillance to monitor the ongoing evolution of antimicrobial resistant determinants and plasmids in Bangladesh and transmission between different countries.

## Introduction

Typhoid and paratyphoid fever are severe systemic infections caused respectively by the human-restricted bacteria *Salmonella enterica* serovar Typhi (*S*. Typhi), and the various *Salmonella* Paratyphi pathovars (*S*. Paratyphi A, B, and C). Collectively, these infections are called enteric fever, with *S*. Typhi accounting for approximately 80% of all enteric fever cases globally. Recent data suggests an increased prevalence of *S*. Paratyphi A (the dominant Paratyphi pathovar) infection in parts of Asia, which has been estimated as ~35% of cases in India and Nepal and >60% of enteric fever in China [1–4]. During a community-based study in densely populated area of Dhaka, Bangladesh, the incidence of *S*. Paratyphi A infections doubled from 0.2 per 1,000 person-years in 2001 to 0.4 episodes per 1,000 person-years in 2004 [5].

*S*. Paratyphi A is recognized as an emerging pathogen and is also commonly reported among travelers returning from endemic regions, as enteric fever vaccines only protect against disease caused by *S*. Typhi [3]. The key antigen of current typhoid conjugate vaccines (TCVs) is the Vi polysaccharide [6]; however, *S*. Paratyphi A lacks the Vi polysaccharide, with the focus of *S*. Paratyphi A vaccine research being the specific O-antigen. A number of promising live attenuated and conjugate *S*. Paratyphi A vaccines are in the early phase of clinical development [2,7]. However, until there is a licensed *S*. Paratyphi A vaccine, or a global improvement in water, sanitation, and hygiene (WASH) conditions, antimicrobial therapy is the only strategy to control paratyphoid fever. Notably, in the case of *S*. Typhi, the widespread use of antimicrobials has resulted in the emergence of antimicrobial resistance (AMR), with a high prevalence of multi-drug resistance (MDR, defined as resistance to ampicillin, chloramphenicol and trimethoprim-sulfamethoxazole) associated with a self-transmissible IncHI1 plasmid, and chromosome-mediated resistance to fluoroquinolones. Correspondingly, there are less data regarding plasmid-mediated AMR in *S*. Paratyphi A, with some reports suggesting a very low prevalence of MDR but an increasing trend of fluoroquinolone resistance due to chromosomal mutations in the DNA gyrase (*gyrA*, *gyrB*) genes and the topoisomerase IV (*parC*, *parE*) genes [4,7–11]. Additionally, apart from the rising trend of fluoroquinolone resistance in *S*. Paratyphi A, there have been sporadic reports of azithromycin resistant isolates [8], and a bla_*CTX*-M-15_ extended-spectrum beta-lactamases (ESBL) producing *S*. Paratyphi A isolate was isolated from a traveler returning to UK from Bangladesh in 2017 [12].

Data regarding the molecular epidemiology, including the population structure and transmission dynamics, of this neglected emergent pathogen is limited from endemic countries like Bangladesh. Whole genome sequencing (WGS) is an excellent tool for generating new insights into bacterial pathogens, and sequencing data will aid in the identification of specific targets for better diagnostics and designing potential vaccine candidates. Here, we exploited WGS to better understand the population of *S*. Paratyphi A isolated from patients in Bangladesh in the last 10 years until 2018. We employed this method to comprehensively assess the genomic variation, AMR determinants, and auxiliary plasmid profiles of 67 *S*. Paratyphi A isolated between 2008 and 2018 from eight enteric disease surveillance study sites in Bangladesh.

## Methods

### Ethics statement

Ethical approval was obtained from the Research Review Committee (RRC) and Ethical Review Committee (ERC) of the International Centre for Diarrhoeal Disease Research, Bangladesh (icddr,b). Informed written consent was taken from adult participants and the legal guardians of child participants under 18 years old for the studies from which isolates were collected.

### Study settings

**Fig 1:**
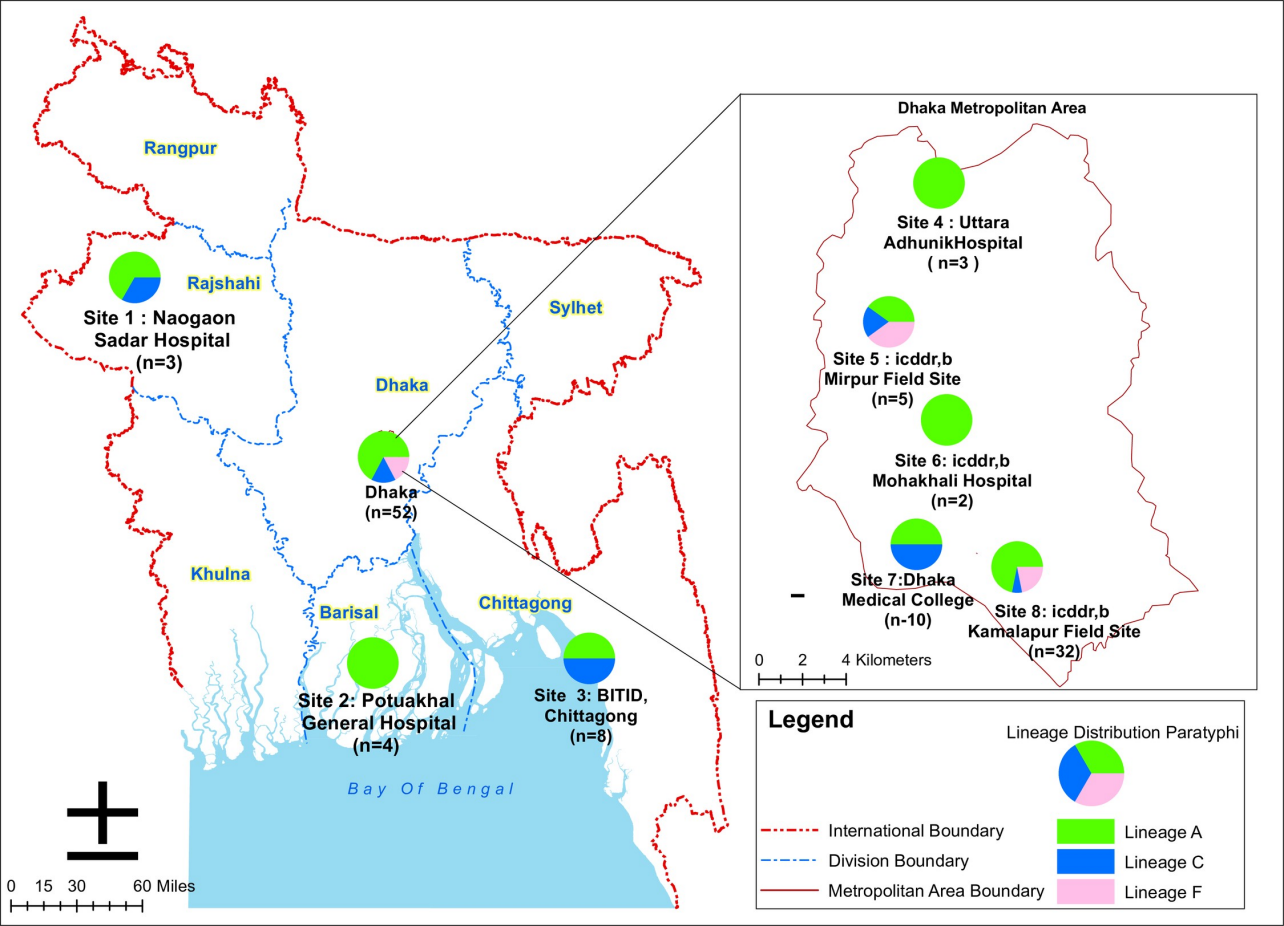
Geographical distrbution of *S*. Paratyphi A lineages in each study site of Bangladesh. Map of Bangladesh showing the lineage distribution at each of the eight study sites across Bangladesh (Direct base layer link for Bangladesh and Dhaka map available athttps://geodash.gov.bd/layers/geonode:level_1_administrative_areas and https://geodash.gov.bd/layers/geonode:dhaka_administrative_boundaries respectively, maps sourced from https://geodash.gov.bd and modified by using ArcGIS10.8.1 software). Pie charts at each site depict the lineage distribution. The number of *S*. Paratyphi A positive cases (*n*) at each study site are also shown in the key.

icddr,b is an international health research organization located at Mohakhali area in Dhaka which contributed to various enteric disease surveillance studies across Bangladesh. We designed this genomic study with eight different enteric disease surveillance study sites in Bangladesh that conducted by icddr,b. A nationwide enteric disease surveillance study [13] was conducted in ten hospitals located in eight different districts of Bangladesh in collaboration with the Institute of Epidemiology, Disease Control and Research (IEDCR), from which we included all the available *S*. Paratyphi A isolated between 2014 and 2018 from five hospitals (Site 1: Naogaon Sadar Hospital, Site 2: Potuakhali General Hospital, Site 3: BITID, Chittagong, Site 4: Uttara Adhunik Hospital, Site 7: Dhaka Medical College) for this genomic study (**Fig 1**). In addition, the Typhoid Immunization Surveillance study (TIS study) [14–16] was conducted within three urban areas in Dhaka city and, we included *S*. Paratyphi A isolated between 2008 and 2016 from these three sites (Site 5: icddr,b Mirpur field site, Site 6: icddr,b Mohakhali Hospital and Site 8: icddr,b Kamalapur field site) (**Fig 1)**. For both surveillance studies, suspected enteric fever patients were enrolled from study sites based on the criteria of fever of at least 38°C for a minimum duration of three days.

### Sample collection and bacterial isolation

Blood samples (3mL for children <5 years of age and 5mL for others) from enteric fever suspected patients were collected upon enrollment, inoculated into blood culture bottle and incubated in automated BacT/Alert machines [14]. All positive blood culture bottles were sub-cultured onto MacConkey agar plates and incubated at 37°C for 18–24 hours. Non-lactose fermenting colony(s) from the MacConkey agar plates were inoculated to identify the *Salmonella* spp. by using standard biochemical tests and candidate *S*. Paratyphi A isolates were further confirmed by serotyping with *Salmonella*-specific O and flagellar H antiserum (Denka Sieken Tokyo, Japan) [14,15]. All *S*. Paratyphi A isolates were stored with 20% glycerol at -70°C for further use. In this study, we included all available stored *S*. Paratyphi A isolates (n = 67) for WGS.

### Whole genome sequencing and SNP calling

Genomic DNA was extracted from the *S*. Paratyphi A isolates using the Wizard Genomic DNA Kit (Promega, Madison, WI, USA) according to the manufacturer’s instructions. Index-tagged paired-end Illumina sequencing libraries, with an insert size of 500 bp, were prepared as previously described [17]. WGS was performed at the Wellcome Sanger Institute using the Illumina Hiseq2500 platform (Illumina, San Diego, CA, USA) to generate 150 bp paired end reads. The read quality of each isolate was screened using FastQC (http://www.bioinformatics.babraham.ac.uk/projects/fastqc). Sequence reads were submitted to the European Nucleotide Archive and a full list of accession numbers with metadata for each isolate is summarized in **S1 Table**. Additionally, 242 global *S*. Paratyphi A sequences were also included in the analysis to provide global context (**S2 Table**) [18].

Paired-end reads were mapped against the reference genome of *S*. Paratyphi A AKU_12607 (accession number: FM200053) using SMALT (v0.7.6) [19] to produce a BAM file. SAMtools (v1.9) [20] was used to identify SNPs with Phred scores above 30 and remove low confidence alleles with consensus base quality ≤20, read depth ≤5 or heterozygous base calls. SNPs located in prophage regions, repetitive sequences or recombinant regions as detected by Gubbins (v2.4.1) [21] were also excluded. Then SNP-sites (v2.5.1) [22] extracted the SNPs from the multi-FASTA alignment and SNP distances were calculated using the dna.dist function in the Analysis of Phylogenetics and Evolution (ape) R package (v5.4–1) [23].

### Phylogenetic and statistical analysis

RAxML (v8.2.8) [24] was used to construct a maximum likelihood (ML) phylogenetic tree from the SNP alignment. A generalized time-reversible model and a gamma distribution was used to model site-specific rate variation (GTR+ Γ substitution model; GTRGAMMA in RAxML) with 100 bootstrap pseudo-replicates used to assess branch support for the ML phylogeny. The resulting phylogenies were visualized and annotated using FigTree (v1.4.4) (http://tree.bio.ed.ac.uk/software/figtree), and the R package ggtree (v2.4.1) [25]. We performed Fisher’s exact tests implemented in STATA [26] to determine statistical differences of lineage distribution between study sites inside and outside Dhaka.

### Comparative pan-genome analysis

Raw sequence reads were assembled *de novo* using Unicycler (v0.4.7) [27] and annotated with Prokka (v1.12) [28]. The quality of genome assemblies were assessed by QUAST (v5.0.2) [29] and the assembly metrics i.e. number of contigs (≥1,000bp) and total assembly length (contigs ≥1,000bp) of both Bangladeshi and global Paratyphi A isolates are summarised in **S3 Table.** We included genome assemblies that meet the threshold less than 100 contigs by using the formula (mean + 0.5 standard deviation) for comparative pan-genome analysis. Roary (v3.12.0) [30] was performed on annotated assembled genomes to identify the pan-genome, using a blastp percentage sequence identity of 95% and a core definition of present in ≥ 95% of the included isolates. The Heaps function within the Micropan R package [31] was used to plot the pan-genome curve which calculates the curve fit constant according to Heaps law [32]: n = k*N^-α^, where n is pan-genome size, N is the number of genomes and k is the curve-specific constant. The exponential term, α determines whether the pan-genome of a bacterial variant is closed (α>1) or open (α<1).

### Antimicrobial resistance gene, plasmid identification and comparative pHCM2 plasmid analysis

ARIBA (v2.14.4) [33], in conjunction with the comprehensive antibiotic resistance database (CARD) [34], and the PlasmidFinder database [35] were used to detect AMR genes and plasmid replicons, respectively. AMR mutations in *S*. Paratyphi A genomes, including point mutations in the quinolone resistance-determining region (QRDR) of genes *gyrA*, *parC* and mutation in *acrB* associated with azithromycin resistance were identified using the genoparatyphi python script (available at: https://github.com/zadyson/genoparatyphi/).

To investigate the genetic diversity, evolution, and circulation of the pHCM2 plasmids present in Bangladeshi *S*. Typhi and *S*. Paratyphi populations, we constructed pHCM2 plasmid phylogeny including 17 *S*. Paratyphi A genomes from this present study and 334 *S*. Typhi and a single *S*. Paratyphi B variant Java (*S*. Java) genome from previous studies in Bangladesh [36–38]. A full list of accession numbers for each genome used for pHCM2 phylogeny is summarized in **S4 Table**. Briefly, all the raw sequencing reads from these organisms were mapped to the reference *S*. Typhi CT18 pHCM2 plasmid (accession number: AL513384.1) using local sensitive mapping Bowtie2 (v2.4.2) [39] and SNPs were identified using SAMTools (v1.3.1) [20]. SNPs that did not meet the quality criteria (Phred score ≥ 30, depth coverage ≥5) were excluded from plasmid analysis. RAxML v8.2 [24] was used for constructing a maximum likelihood (ML) plasmid phylogeny based on the total of 1788 core SNPs calling from mapping. RAxML (v8.2.8) used the generalized time-reversible evolutionary model with gamma-distributed rate variation (GTR+ Γ). One hundred bootstrap pseudo-replicate analyses were performed to assess the robustness of the ML tree topology. Moreover, we also compared the annotated pHCM2 plasmid sequence of *S*. Paratyphi A and *S*. Paratyphi B variant Java and to the reference pHCM2 plasmid by BLAST [40] to identify sequence similarity with the database and visualized these comparisons using Artemis Comparison Tool (ACT) (v18.0.2) [41] and Easyfig (available at https://mjsull.github.io/Easyfig/). pHCM2 plasmid integrity was confirmed by visualizing the assembly graph of pHCM2 plasmid carrying *S*. Paratyphi A genome in assembly graph viewer Bandage tool (v0.8.1) (available at https://rrwick.github.io/Bandage/).

## Results

### The population structure of *S*. Paratyphi A isolates in Bangladesh

**Fig 2:**
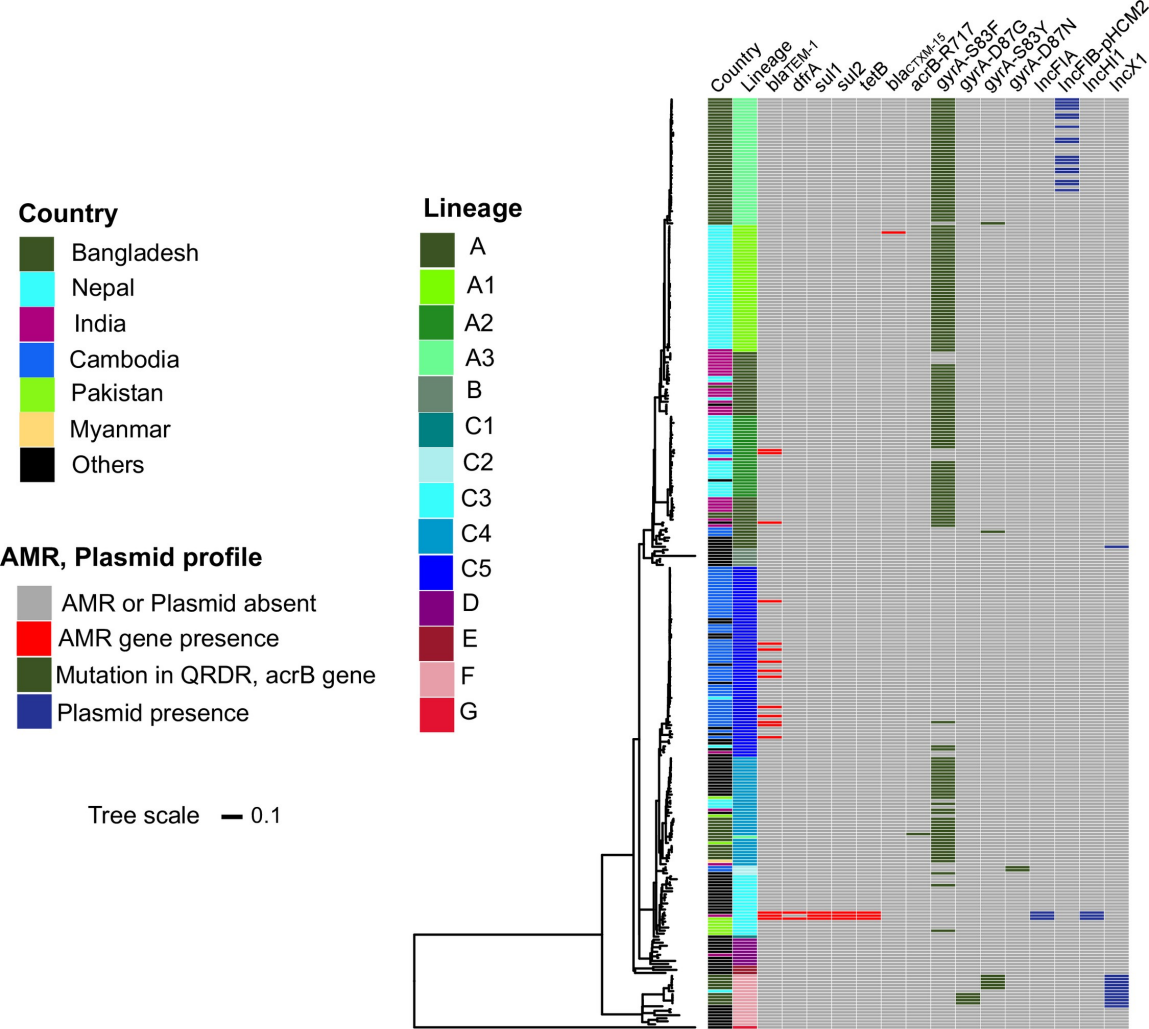
Global population structure of Bangladesh *S*. Paratyphi A with AMR genes and plasmid replicons presence heatmap. Maximum-likelihood phylogenetic tree of 309 *S*. Paratyphi A genome sequences from the global collection including Bangladeshi isolates from this study. The coloured heatmap shows country, lineages, AMR genes, mutations in the QRDR and *acrB* genes, plasmid replicons for each isolate; see legend for colours.

We subjected the genome sequences of the 67 Bangladeshi *S*. Paratyphi A isolates and an additional 242 global *S*. Paratyphi A genome sequences to phylogenetic analysis. We obtained a final set of 5,419 chromosomal SNPs from a total alignment length of 4,794,508 bp for the total 309 *S*. Paratyphi A genomes. The resulting global phylogeny could be subdivided into the seven previously defined distinct lineages (A to G); the *S*. Paratyphi A isolated from Bangladesh were restricted to only lineages A (67.2%), C (19.4%) and F (13.4%) (**Fig 2**). *S*. Paratyphi A isolates in this study were most closely related to isolates originating in Nepal, India, Pakistan, and Myanmar, suggesting regional circulation of these lineages across South Asia. Notably, the majority (42/67, 62.6%) of the contemporary Bangladeshi sequences formed a monophyletic sub-lineage within lineage A that we herein defined as sub-lineage A3. This dominant Bangladeshi sub-lineage was closely related to sub-lineage A1 isolates from Nepal (median distance ~70 SNPs). Moreover, three Bangladeshi isolates were intermingled with lineage A Indian isolates on two independent branches separated by median of 25 and 9 SNPs respectively. Aside from lineage A, a single Nepalese *S*. Paratyphi A isolate from lineage F was grouped within nine Bangladeshi isolates and separated by median of 44 SNPs. The remaining 13 isolates clustered within sub-lineage C4 as did organisms from Pakistan and Myanmar (median distance 57 SNPs).

We additionally assessed the distribution of lineage A, C, and F among 67 isolates collected from eight study sites covering four divisions within Bangladesh (**Fig 1**). This geographical location data outlined a heterogeneous distribution of lineages between study sites, with the exception of some study sites with a limited number of isolates. Lineage A was distributed throughout all eight study sites, with three sites (2, 4, and 6) harboring isolates from lineage A only. All these three lineages (A, C, F) were detected in the icddr,b Kamalapur field site (site 8; n = 32) and the Mirpur field sites (site 5; n = 5), located in Dhaka. Two lineages (A, C) were restricted to the remaining three hospital sites (1, 3, and 7). We did not observe any significant difference (*p* = 0.15; Fisher’s exact test) in the distribution of lineages between the five study sites within Dhaka (sites 4, 5, 6, and 7; n = 52) and the three study sites outside Dhaka (sites 1, 2, and 3; n = 15).

### Comparative pan-genome analysis

We first conducted a pan-genome analysis on all 309 *S*. Paratyphi A genomes to investigate the gene distribution among the serovar. The pan-genome comprised 18,802 genes including 4,054 core genes (detected in ≥ 95% of all genomes) and 14,748 accessory genes (detected in < 95% of all genomes). The seemingly overestimated accessory gene set, driven by the presence of 14,748 cloud genes (detected in only < 15% of total genome), was due to fragmentation of genome assemblies. To minimize the effect of low-quality sequence data, we further assessed the quality of genome assemblies (i.e explained details in method; **S3 Table)**, and reconsidered the comparative pan-genome analysis using 295 quality-filtered assemblies. The revised pan-genome contained 9,093 genes, divided into 4,125 core genes and 4,968 accessory genes. In a typical *S*. Paratyphi A genome (average 4,300 coding sequences, n = 295), the core genes account for 96.1% of the coding sequences. We also observed the gene accumulation curve of *S*. Paratyphi A pan-genome (n = 295) slowly flattened with the addition of new genomes, with a curve fitting parameter α value of 1.000015, indicating a closed pan-genome (**S1 Fig**). Based on the pan-genome size and the proportion of a typical genome comprised of core genes, we concluded that the *S*. Paratyphi A possesses a conservative genomic structure with little evidence of importation of new genes.

### AMR genes and plasmid profiles associated with Bangladeshi *S*. Paratyphi A

We explored the Bangladeshi *S*. Paratyphi A genome sequences together with a global collection of *S*. Paratyphi A from previous studies for genes and mutations associated with AMR and also to identify plasmid replicons (**Fig 2**). Notably, and contrary to previously published data from *S*. Typhi from Bangladesh, the AMR genes, *catA*, *dfrA7*, *sul1*, *sul2*, *strA*, *strB*, and *bla*_*TEM-1*_, which confer an MDR phenotype were not detected in our Paratyphi A collection. Despite a lack of genes associated with MDR, non-synonymous mutations in the QRDR (either codon 83 or 87) of *gyrA*, associated with reduced susceptibility to fluoroquinolones, were found in all 67 of the Bangladeshi *S*. Paratyphi A isolates. Among the *gyrA* QRDR mutations detected, S83F (85.1%; 57/67) was the most common, followed by S83Y (9%; 6/67), and D87G (6%; 4/67). No double or triple QRDR mutations in *gyrA*, *gyrB*, *parC*, *parE* genes were observed in any *S*. Paratyphi A isolates of this study. In addition, a single *S*. Paratyphi A organism isolated from Dhaka Medical College (site 7) in 2018 carried an R717L mutation in *acrB*, which conferred resistance to azithromycin. Plasmid associated sequences were limited in these genome sequences, an IncX1 plasmid and IncFIB-pHCM2-like plasmid were identified in 12 and 17 isolates, respectively (**Fig 2**).

### The origins of pHCMC2-like plasmid in Bangladeshi *S*. Paratyphi A

**Fig 3:**
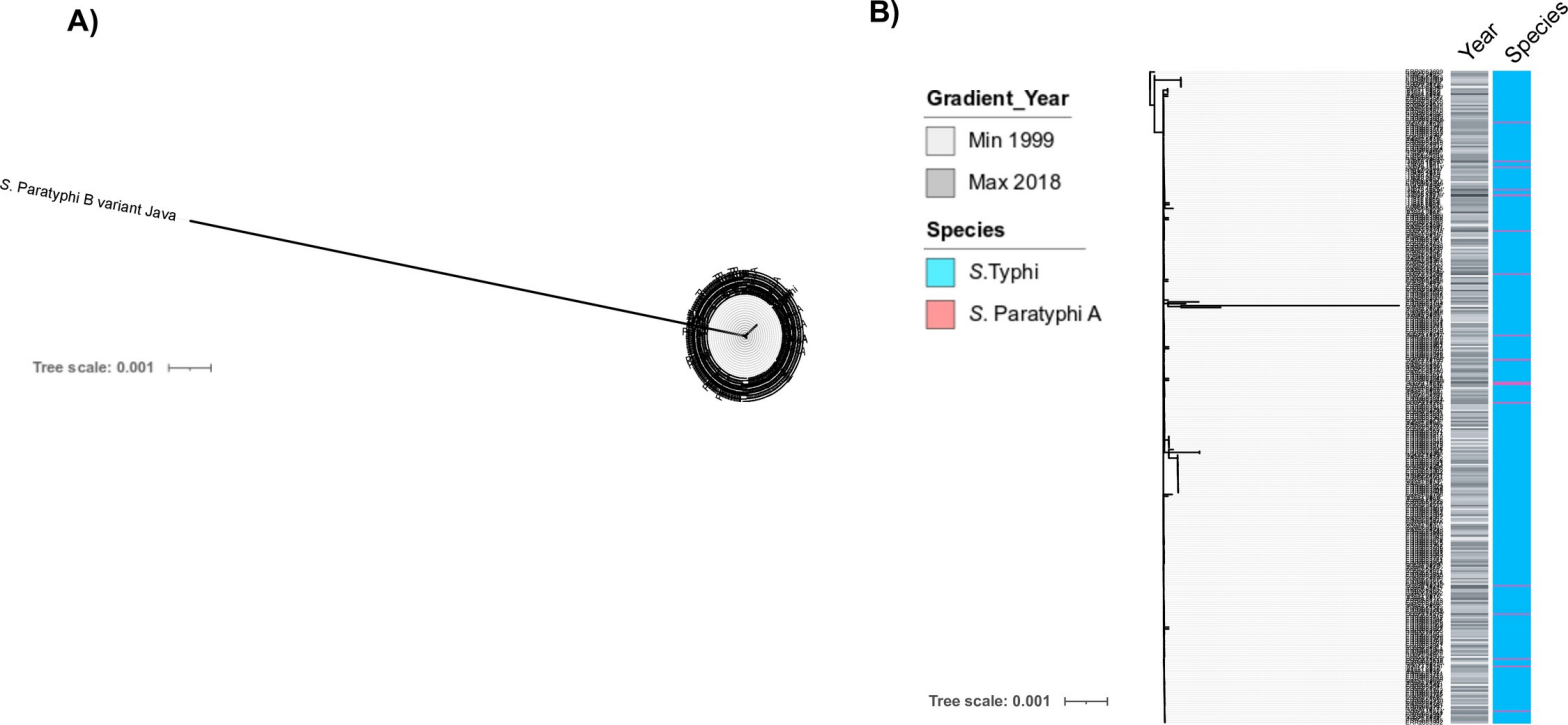
The local evolutionary history of the pHCM2 plasmid in Bangladesh. (A) Unrooted of maximum likelihood whole pHCM2 phylogeny including *S*. Paratyphi A isolates from this study and *S*. Typhi, *S*. Paratyphi B variant Java from previous studies in Bangladesh (B) Maximum likelihood of pHCM2 phylogeny including only *S*. Paratyphi A and Typhi isolates. The coloured heatmap shows species and year of collection for each isolate.

Aiming to better understand the emergence of pHCM2-like plasmid in 17 Bangladeshi *S*. Paratyphi A isolated from this present study, we conducted further fine-detailed comparisons of these genomes with the reference *S*. Typhi CT18 pHCM2 plasmid and a non-typhoidal *Salmonella* serovar (*S*. Java). We constructed a pHCM2 phylogenetic tree incorporating plasmid sequences from the 17 *S*. Paratyphi A, 334 *S*. Typhi, and one *S*. Java isolated from Bangladesh (**Fig 3 and S4 Table**) to understand the recent evolutionary history of this cryptic pHCM2 plasmid. The pHCM2 plasmid phylogeny and BLAST analysis revealed that the pHCM2 sequences from *S*. Typhi (106,516 bp) and *S*. Paratyphi A (106,706 bp) were highly related, with 99.98% nucleotide identity (average 19 SNPs variation). Importantly, the *S*. Paratyphi A pHCM2- like plasmid assembled into a single contig that was predicted to be circular by analysis using Bandage (S2 Fig). Conversely, a more distantly related pHCM2-like plasmid has been previously detected in non-typhoidal *S*. Java (107,362 bp), and shares 98.8% identity with *S*. Typhi CT18 pHCM2 plasmid across only 90% of the sequence (average 1,697 SNPs variation).

Next, we compared the annotated pHCM2 reference plasmid sequence of *S*. Typhi CT18 with *S*. Paratyphi A and *S*. Java to identify the genetic difference of pHCM2 between typhoidal and non-typhoidal *Salmonella* and identified 12 non-identical regions against pHCM2 plasmid reference, including three insertions and one deletion (**S2 Fig, S5 Table**). Most of these non-identical regions were hypothetical proteins and only a few were related to DNA metabolism and phage. Furthermore, we conducted pan-genome analysis on all 309 *S*. Paratyphi A and identified 127 proteins (113 hypothetical proteins and 14 annotated proteins) which were present in only the 17 pHCM2-like plasmid-harboring Bangladeshi *S*. Paratyphi A isolates but not present in other global *S*. Paratyphi A isolates (**S6 Table).**

## Discussion

In this study, we performed whole genome sequencing of *S*. Paratyphi A collection isolated during nationwide enteric disease surveillance in Bangladesh and combined these sequence data with an additional 242 sequences from a global collection of *S*. Paratyphi A genomes [18]. Previously, a global population structure study by Zhou *et al*. 2018 classified *S*. Paratyphi A into seven lineages and identified lineages A and C as the most dominant globally [42]. Our phylogenetic analysis confirmed these data, finding lineage A to be the most common in Bangladesh, with the overall population comprised of only three lineages (A, C, and F). The addition of 67 Bangladeshi *S*. Paratyphi A isolates into the global phylogeny suggests that there may have been an ongoing clonal expansion of lineage A. This lineage appears to have been imported from neighboring countries on multiple occasions, which contrasts with onservations from lineages C and F which appeare to have been introduced on one occasion in this dataset, all of which provides information to track ongoing local or international transmission for surveillance efforts. Additionally, the apparently random distribution of these three lineages across eight nationwide surveillance sites provides limited evidence for regional geographic restriction of *S*. Paratyphi A lineages in Bangladesh.

Over the past two decades, the emergence of resistance to the first line antimicrobials for *S*. Typhi infections has led to change in treatment. Firstly, fluoroquinolones were adopted, followed by third generation cephalosporins and the antibiotic azithromycin. High incidence of MDR typhoid, associated with self-transmissible IncHI1 plasmids, has been previously reported in many endemic settings throughout Asia and Africa [18,37,43]. Notably, the absence of MDR genes in the genome sequences of these 67 Bangladeshi *S*. Paratyphi A isolates is in stark contrast with *S*. Typhi. Indeed, studies from Nepal and Pakistan have identified cases of *S*. Paratyphi A associated with MDR phenotypes and resistance to third-generation cephalosporins mediated by extended spectrum beta lactamases [44,45]. The potential exception to the surprising lack of AMR, was the resistance (or reduced susceptibility) to fluoroquinolones. Decreased fluoroquinolone susceptibility is associated with chromosomal mutations in the QRDR. Our data shows that single chromosomal *gyrA* point mutations in QRDR was observed among all Bangladeshi *S*. Paratyphi A isolates. In addition to QRDR mutation, one recent acquisition of R717L mutation in *acrB* gene associated with azithromycin isolated from Dhaka in 2018 is of major concern. Similar mutations have been recently identified in *S*. Typhi and *S*. Paratyphi A in Nepal, India, Pakistan and also Bangladesh [8,46,47]. This observation reminds us of the need for constant review of empirical antimicrobials used for the treatment of enteric fever and highlights the need of continued genomic surveillance to monitor ongoing AMR trends in invasive *Salmonella*.

We additionally observed non-AMR associated IncFIB-pHCM2-like cryptic plasmids and IncX1 plasmids among the contemporary Bangladeshi *S*. Paratyphi A isolates, which have also been previously reported in the *S*. Typhi population [36,37], although the pHCM2-like plamid was not detected in any global *S*. Paratyphi A isolates [18]. The abundance of this cryptic pHCM2 plasmid among Bangladeshi *S*. Typhi and *S*. Paratyphi A population has still not been well understood. To our knowledge this is the first time that a pHCM2-like plasmid has been described in *S*. Paratyphi A and our phylogenetic analysis might suggest that the ancestral origins of pHCM2-like plasmid in the *S*. Paratyphi A isolates were likely in *S*. Typhi, and potentially entered into the *S*. Paratyphi A population as early as 2011; the earliest origination of pHCM2 in *S*. Typhi in Bangladesh has been reported in 1999 [36,37]. However, these 17 Bangladeshi *S*. Paratyphi A isolates were only from Dhaka city and restricted within only sub-lineage A3 which indicate that they may have been transferred on a single occasion. A previous study reported that pHCM2 in *S*. Typhi CT18 shared 56% sequence identity with the virulence-associated pMT1 plasmid in *Yersinia* pestis [48]. Here we determined that the pHCM2 sequences from the Bangladeshi *S*. Paratyphi A, *S*. Typhi, and *S*. Java shared approximately 51–59% sequence identity and 96% coverage with pMT1. pHCM2 carries bacteriophage genes and genes related to DNA metabolism and replication. It has been suggested that *Salmonella* enterica serovar Typhimurium rough strain-specific phage SSU5 (SSU5), may be an ancestral form of pHCM2 [49]. Here we found the pHCM2 plasmid shared 81–82% sequence similarity with SSU5 phage genes. We hypothesize that these phage gene regions may have a role in horizontal gene transfer and facilitate pathogen adaptation [50]. Though our pan-genome analysis concluded the *S*. Paratyphi A genome was highly conserved in nature, the presence of the pHCM2-like plasmid in Bangladeshi Paratyphi A expanded the known accessory genome, which might play a role in evolution of this pathogen. The pHCM2 cryptic plasmid was first identified in *S*. Typhi isolates associated with a typhoid case in Vietnam in 1992, before it was subsequently detected in Hong Kong, Cambodia, and Pakistan [51]. The lack of pHCM2-like plasmid in the global collection of *S*. Paratyphi A isolates emphasize our point of interest to understand the source of origin and genetic difference of this plasmid from other *Salmonella* serovars previously detected in Bangladesh. Comparative analysis revealed that pHCM2 from *S*. Paratyphi A and *S*. Typhi shared a higher degree of genetic similarity than with that from *S*. Java. Further studies are required to monitor the trend over time and to understand the physiological effect of this cryptic plasmid on enteric infection caused by *S*. Typhi and *S*. Paratyphi A.

Our study has some limitations. The *S*. Paratyphi A genomes sequenced in this present study were collected from only four out of eight divisions of Bangladesh which may not be representative of the overall population structure of Bangladesh. In addition, we were unable to reach into any statistical significance of the lineage distribution over study sites due to the low number of sequence data generated from this study. However, these data improve our knowledge regarding genomic variation in *S*. Paratyphi A and potential genetic interplay with *S*. Typhi.

*S*. Paratyphi A is an emerging pathogen and may become the leading cause of enteric fever in Asia if typhoid vaccination is introduced in low and middle income countries (LMICs). Therefore, we need a better understanding of the circulation of this pathogen and to investigate if new vaccine approaches are warranted. In the absence of vaccines, monitoring AMR trends, and their associated mechanisms, is important in planning future approaches to therapy for enteric fever. Our data highlight the importance of sustained genome-based surveillance for emerging enteric pathogens like *S*. Paratyphi A in endemic regions.

## Supporting information

S1 Fig Pan-genome accumulation curve of *S*. Paratyphi. **A:** The gene accumulation curve with curve fitting parameter α value of 1.000015 are depicted for global *S*. Paratyphi A genomes (n = 295). Error bars above and below the median are depicted by a vertical line above and below the curve.
(TIF)

S2 Fig Comparison of pHCM2 plasmid reference genome (AL513383) with pHCM2-like plasmid of *S*. Paratyphi A and *S*. Java. (A) The assembly graph of one representative pHCM2-like plasmid harbouring *S*. Paratyphi A genome was visualised in Bandage tool. Each grey line in assembly graph represents a node or assembled contig and a closed ring of pHCM2 plasmid region presented in single node is highlighted as blue colour which was analysed from Bandage’s integrated BLAST search with reference *S*. Typhi CT18 pHCM2 plasmid. (B) Full pHCM2 plasmid sequence comparison of reference AL513384.1 with *S*. Paratyphi A and *S*. Java in Artemis Comparison Tool (ACT) and Easyfig including (C) three insertions and one deletion event in *S*. Paratyphi A relative to pHCM2 plasmid reference. Orange arrows indicted CDSs and grey shading between the sequences represents BLAST nucleotide identity (see key).
(TIF)

S1 Table Metadata including accession numbers, AMR and plasmid profile of 67 *S*. Paratyphi A isolates of this present study. (XLSX)

S2 Table Metadata for the 242 global *S*. Paratyphi A genome collection from previous studies. (XLSX)

S3 Table Genome assembly metrics of total 309 Paratyphi A including *S*. Paratyphi A isolates of this present study. (XLSX)

S4 Table List of lane accession numbers of *S*. Typhi and *S*. Java isolates carrying pHCM2 from previous studies in Bangladesh. (XLSX)

S5 Table Gene list of non-identical regions of annotated pHCM2 plasmid sequence of *S*. Paratyphi A and *S*. Java isolated from Bangladesh and to the reference pHCM2 plasmid. (XLSX)

S6 Table List of unique genes including annotated proteins present in 17 Bangladeshi *S*. Paratyphi A isolates carrying pHCM2-like plasmid but not present in other global Paratyphi A isolates. (XLSX)
