# Supplementary figures and images for: Genetic diversity of Salmonella Paratyphi A isolated from enteric fever patients in Bangladesh from 2008 to 2018
Source: PLoS Negl Trop Dis. 2021 Oct 14;15(10):e0009748. doi: 10.1371/journal.pntd.0009748 (PMC8516307; doi:10.1371/journal.pntd.0009748)

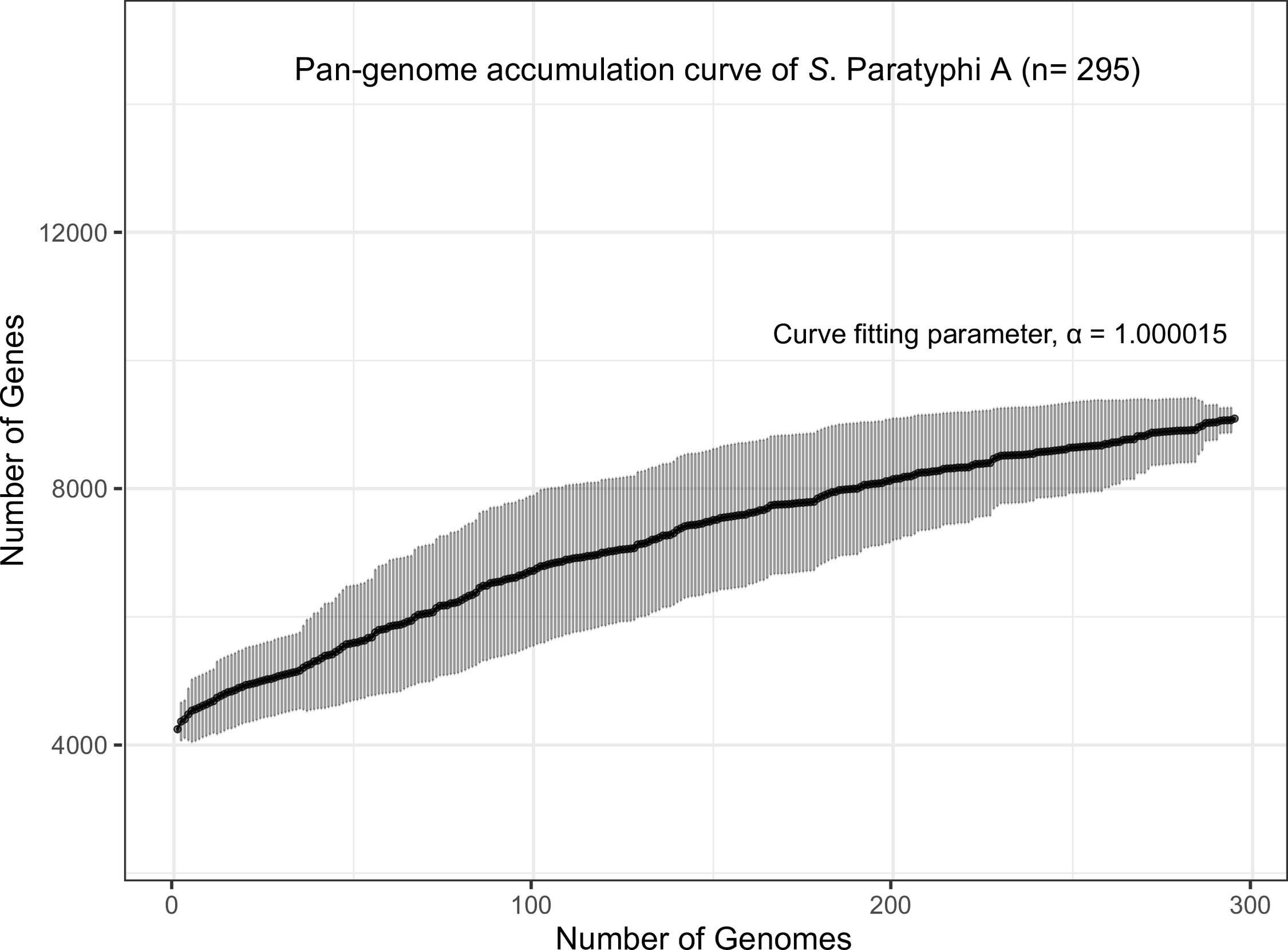

Supplement: S1 Fig — A: The gene accumulation curve with curve fitting parameter α value of 1.000015 are depicted for global S. Paratyphi A genomes (n = 295). Error bars above and below the median are depicted by a vertical line above and below the curve. (TIF) [file pntd.0009748.s001.tif]

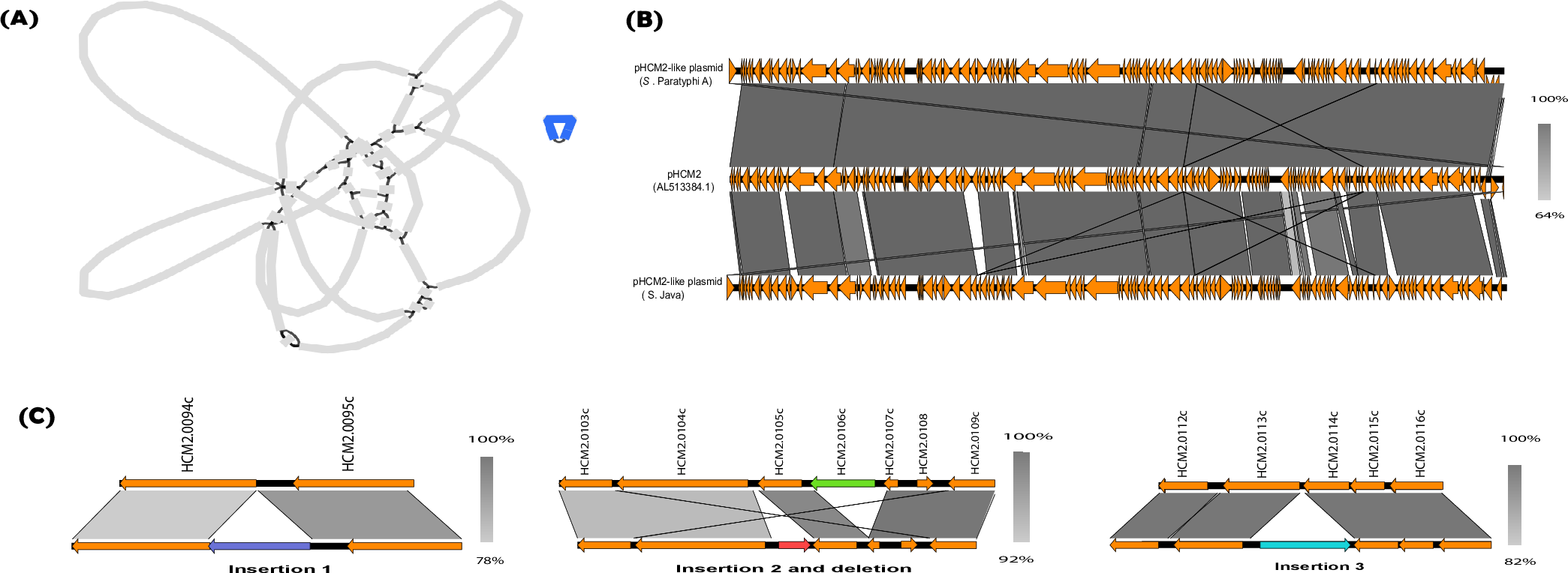

Supplement: S2 Fig — (A) The assembly graph of one representative pHCM2-like plasmid harbouring S. Paratyphi A genome was visualised in Bandage tool. Each grey line in assembly graph represents a node or assembled contig and a closed ring of pHCM2 plasmid region presented in single node is highlighted as blue colour which was analysed from Bandage’s integrated BLAST search with reference S. Typhi CT18 pHCM2 plasmid. (B) Full pHCM2 plasmid sequence comparison of reference AL513384.1 with S. Paratyphi A and S. Java in Artemis Comparison Tool (ACT) and Easyfig including (C) three insertions and one deletion event in S. Paratyphi A relative to pHCM2 plasmid reference. Orange arrows indicted CDSs and grey shading between the sequences represents BLAST nucleotide identity (see key). (TIF) [file pntd.0009748.s002.tif]
